# Influence of attentional state on EEG-based motor imagery of lower limb

**DOI:** 10.3389/fnhum.2025.1545492

**Published:** 2025-05-21

**Authors:** Penghai Li, Dongfang Yu, Longlong Cheng, Kun Wang

**Affiliations:** ^1^School of Electrical Engineering and Electronics, Tianjin University of Technology, Tianjin, China; ^2^China Electronics Cloud Brain (Tianjin) Technology Co., Ltd., Tianjin, China; ^3^Haihe Laboratory of Brain-Computer Interaction and Human-Computer Integration, Tianjin, China; ^4^School of Medicine, Tianjin University, Tianjin, China

**Keywords:** attentional state, motor imagery, ERD, brain–computer interface, theta/beta ratio

## Abstract

**Introduction:**

Motor imagery (MI) has emerged as a promising technique for enhancing motor skill acquisition and facilitating neural adaptation training. Attention plays a key role in regulating the neural mechanisms underlying MI. This study aims to investigate how attentional states modulate EEG-based lower-limb motor imagery performance by influencing event-related desynchronization (ERD) and the alpha modulation index (AMI) and to develop a real-time attention monitoring method based on the theta/beta ratio (TBR).

**Methods:**

Fourteen healthy right-handed subjects (aged 21–23) performed right-leg MI tasks, while their attentional states were modulated via a key-press paradigm. EEG signals were recorded using a 32-channel system and preprocessed with independent component analysis (ICA) to remove artifacts. Attentional states were quantified using both the traditional offline AMI and the real-time TBR index, with time–frequency analysis applied to assess ERD and its relationship with attention.

**Results:**

The results indicated a significant increase in ERD during high attentional states compared to low attentional states, with AMI values showing a strong positive correlation with ERD (*r* = 0.9641, *p* < 0.01). Cluster-based permutation testing confirmed that this *α*-band ERD difference was significant (corrected *p* < 0.05). Moreover, the TBR index proved to be an effective real-time metric, decreasing significantly under focused attention. Offline paired *t*-tests showed a significant TBR reduction [*t*_(13)_ = 5.12, *p* = 2.4 × 10^−5^], and online analyses further validated second-by-second discrimination (Bonferroni-corrected *p* < 0.01). These findings confirm the feasibility and efficacy of TBR for real-time attention monitoring and suggest that enhanced attentional focus during lower-limb MI can improve signal quality and overall performance.

**Conclusion:**

This study reveals that attentional states significantly influence the neural efficiency of lower-limb motor imagery by modulating ERD/AMI and demonstrates that the TBR can serve as a real-time indicator of attention, providing a novel tool for optimizing attentional processes in motor skill training.

## 1 Introduction

Motor imagery (MI), a technique that enables mental simulation of movements without actual execution, has been shown to play a significant role in motor skill learning (Burianová et al., 2013), performance enhancement (Guillot et al., 2010), and motor control. Studies (Jeunet et al., 2020) have demonstrated that athletes’ attention abilities are positively correlated with resting-state alpha power. Research in motor neuroscience indicates that cognitive processes—particularly attention—play a critical role in modulating the neural substrates associated with MI. Numerous studies (Gabbard and Fox, 2013; Nicklas et al., 2024) have revealed that heightened attentional states can improve the quality of motor imagery (Zhang et al., 2016; Sakurada et al., 2016), as evidenced by event-related desynchronization (ERD) (Groppe et al., 2013) and other neurophysiological indicators, such as the alpha modulation index (AMI) (Evans and Abarbanel, 1999).

## 2 Related work

In the realm of MI, attentional focus is recognized as a critical determinant of performance (Clark et al., 2015). McNevin et al. (2000) demonstrated that high levels of attention are essential for optimal motor skill acquisition, while research by Hall (2009) and Collet et al. (2011) indicated that appropriately paced MI can modulate actual motor performance, allowing athletes to make more effective corrections during movement. Recent advancements have further elucidated the role of attention in MI and motor control. Quadrato Motor Training (QMT) has emerged as an innovative form of movement meditation designed to enhance creativity and reflective capacity through the modulation of alpha activity (Ben-Soussan et al., 2014). Concurrently, studies by Clark et al. (2015) and Posner et al. (2015) have underscored that mindful movement practices and attention training not only improve motor control but also mitigate lapses in cognitive focus. Neurofeedback interventions, as reviewed by Jiang et al. (2017), provide additional evidence that training can enhance attention and memory, thereby preventing cognitive decline.

The relationship between neural oscillations and attentional states has been investigated for nearly a century, with Berger’s seminal work (Berger, 1929) establishing a link between alpha band activity and attention/arousal levels. Subsequent studies, such as those by Klimesch et al. (1998), have confirmed that variations in alpha power, accompanied by corresponding changes in delta activity, serve as reliable indices of individual attentional engagement. Moreover, motor imagery is closely related to various neurophysiological indices (e.g., alpha, theta, and beta waves), which reflect verbal processing, conscious cognitive operations (Jeunet et al., 2020), and covert visuo-spatial attention during motor skill acquisition (Parr et al., 2023).

Currently, most research has concentrated on upper limb (Cioffi et al., 2024) motor imagery (Lee et al., 2016) or its application in stroke rehabilitation (Ma et al., 2024). However, investigations into real-time attention monitoring during motor skill training and neural adaptation training in healthy adults, especially athletes, remain relatively scarce (Grilc et al., 2024). Traditional attention assessment methods such as the AMI, although effective in offline conditions, are inadequate for meeting the rapid feedback demands of motor training (Jeunet et al., 2020). To address this gap, the present study introduces the TBR as a real-time attention tracking index. Its high temporal resolution makes it a powerful tool for online monitoring of attentional states (van Son et al., 2019). Collectively, these studies provide a robust framework for understanding the neural underpinnings of attentional control in MI and motor performance.

The aim of this study is to develop and validate a novel EEG-based real-time attention tracking method that employs the TBR to investigate how different attentional states (high vs. low) modulate neural representations during lower-limb motor imagery in healthy adults by assessing neurophysiological indicators such as ERD and the AMI, thereby providing valuable insights for optimizing motor training protocols and advancing our understanding of cognitive–motor interactions.

## 3 Materials and methods

### 3.1 Experiment subjects

The experiment was performed in the Lab of EEG Acquisition and Application, Tianjin University of Technology, China. These EEG experiments were approved by the Ethics Committee. The subjects in this experiment were 14 young volunteers (7 male and 7 female volunteers) who were physically and mentally healthy and had no mental illness in the past. The subjects were 21 to 23 years old, all right-handed, and none of them had experience in EEG experiment. The subjects had been informed of the purpose and precautions of the experiment. The purpose of the experiment is to collect the EEG signals during the subjects’ motor imagination for processing and analysis. These data are only used for the scientific research of this article and are not used for other purposes. Precautions of these experiments: There is a rest time between each trial. During that time, the subject relaxes the body as much as possible. When the next trial starts, the subjects should try not to carry out muscle activities as much as possible to reduce the interference of muscle activities on EEG signals. They can choose not to watch the video after they feel that they will perform motor imagery, to prevent the subjects from only performing action observation. Then, they signed the experimentally informed consent before the experiment.

### 3.2 Experiment paradigm

During the experiment, each subject was asked to sit in a comfortable chair with a computer screen about 50 cm away in front of his/her eyes. The experimental paradigm was designed with Python.

**Figure 1:**
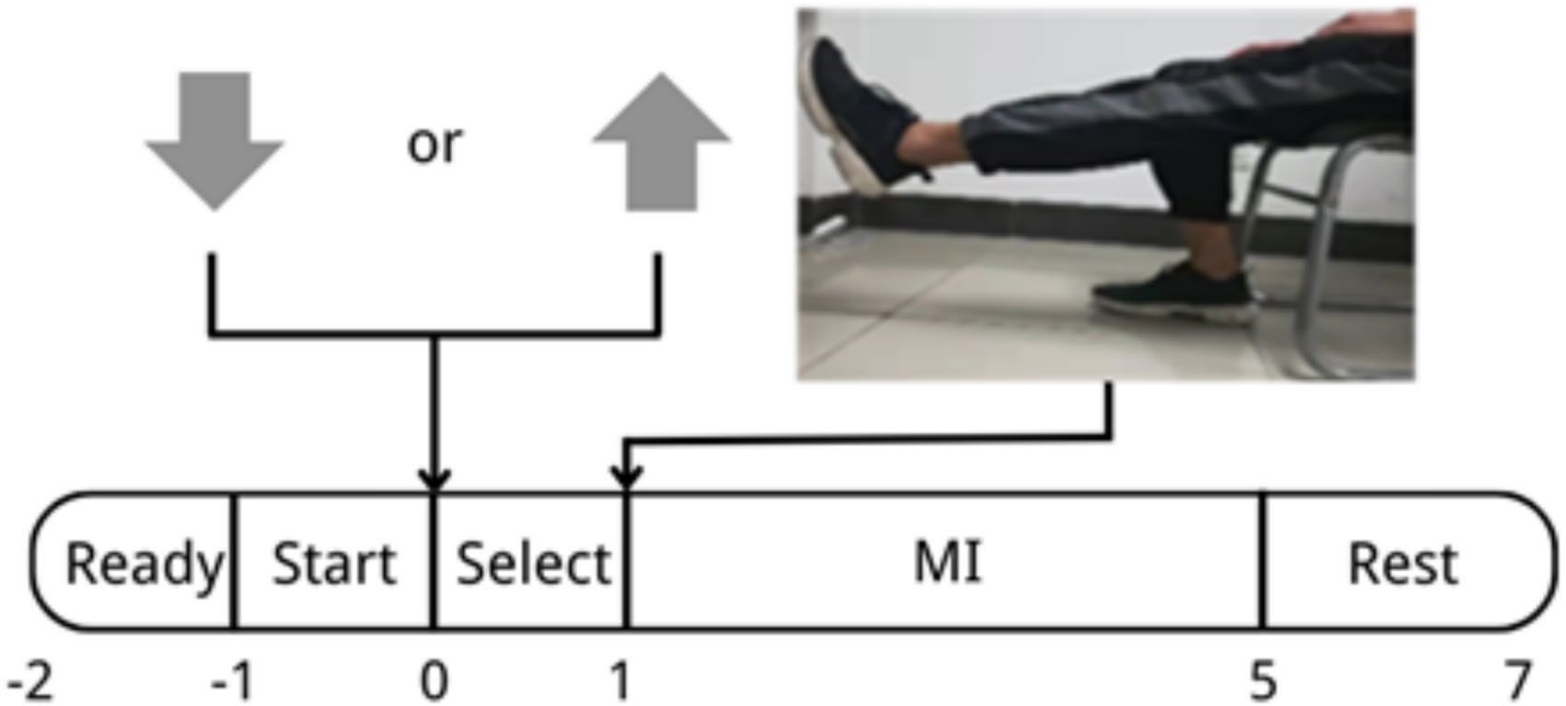
Experimental paradigm.

The experiment process is shown in Figure 1. During the −2–−1 s phase, the word “Ready” appeared on the display screen. This process lasted for 1 s, which meant that the experiment would start immediately and the subject would remain resting. At 1–0 s, the word “Start” appeared on the screen, giving the subject a hint for preparation to start soon. At 0–1 s, the computer screen showed “Select”; at this moment, an arrow pointing up or down would randomly appear on the screen. The judgment rule was as follows: An up-pointing or down-pointing arrow randomly appeared on the selection interface, and these two types of arrows appeared with the same probability, each accounting for 50%. The subject responded by pressing the corresponding (up arrow or down arrow) key as indicated by the arrow on the screen. The key pressing process is used to improve the subjects’ alertness; meanwhile, the correctness was used to judge whether the subject had a high or low level of attention (Attention or Inattention). Because the research focus of this study is the improvement of the effect of motor imagination after the execution of “select,” not the change of attention during the execution of the arrow decision task, when designing the paradigm, it is considered that on the basis of reducing the impact of the paradigm on motor imagination as much as possible, the subjects can also focus their attention to a certain extent. After comprehensive consideration, the arrow decision task is adopted. During 1–5 s, the screen showed a video which lasted for 4 s. During this period, the subject followed the guided video to imagine the movement of the right leg. In 5–7 s, the word “Rest” appeared on the screen, allowing the subject to relax and set the brain into a resting state, which indicated that the experiment of one trial was over. For each subject, the experiment was conducted in 4 sets, each including 20 trials, and the subjects rested for 5 min between two sets to avoid negative effect on the experimental result due to fatigue. In the experiments mentioned below, this paradigm is applied in offline and online experiments.

### 3.3 EEG data acquisition and preprocessing

The experimental equipment Grael is an electrophysiological amplifier developed by Compumedics company in Australia. It has 32 channels, and the electrode position is set in the international 10/20 system. The collected EEG raw data were preprocessed by first resampling from 1,000 to 200 Hz and applying a band-pass filter (1–30 Hz) to remove power frequency interference. Subsequently, artifacts due to subject activity—including ocular movements, muscle artifacts, and other noise—were removed using independent component analysis (ICA) (Burianová et al., 2013). We applied ICA to decompose the raw EEG data into independent components. Each component was visually inspected based on its time course, spectral properties, and scalp topography. Components exhibiting characteristic artifacts—such as ocular movements, muscle activity, or electrical noise—were automatically identified using the ADJUST algorithm (Mognon et al., 2011). Only components that met the criteria were rejected, ensuring that only artifact-related activity was removed. After the rejection process, the remaining components were transformed back into the electrode space using the inverse of the ICA mixing matrix, thereby reconstructing the artifact-free EEG signals. In this study, FP1 and FP2 leads are mainly used to eliminate the impact of blinking on the data. The baseline is the data of resting state (1 s before stimulation, i.e., −1–0 s), and the baseline correction is completed in the preprocessing stage.

### 3.4 Statistical methods

All statistical analyses in this study were conducted using Python programming with open-source scientific libraries, ensuring transparency and reproducibility. Below, we describe in detail each statistical method and associated parameters for the analyses performed.

Event-related spectral perturbation (ERSP) was calculated to quantify EEG spectral changes during motor imagery MI tasks. The ERSP values were computed using the formula:


(1) $$\text{ERSP}(f,t)=\frac{1}{N}\sum_{k=1}^{N}|K(f,t,k)|2$$

where N denotes the total number of trials, and K(f,t,k) represents the spectral estimate at frequency f and time t for the $k^{\mathrm{th}}$ trial. Spectral estimates were obtained using Morlet wavelet transforms, implemented with the “Waveletpacket()” function from the PyWavelets library (pywt). The chosen mother wavelet was “db4” with a decomposition level of 8, yielding a frequency resolution covering 1–30 Hz. ERSP values were computed within a time window from −1 to 5 s, relative to the cue onset. ERD was quantified specifically within the alpha (8–13 Hz) frequency band and time period (1–5 s) corresponding to the MI phase. ERD was calculated according to following formula:


(2) $$\mathrm{ERD}=\frac{1}{M}\sum_{f=f_{1}}^{f_{2}}\sum_{t=t_{1}}^{t_{2}}(\text{ERSP}(f,t))$$

where $f_{1}$ and $f_{2}$ define the alpha frequency range, while $t_{1}$ and $t_{2}$ denote the MI task time interval. ERD values provided an averaged measure of cortical desynchronization across trials and subjects.

To quantify attentional states, the alpha modulation index (AMI) was computed based on FFT-derived alpha power from EEG signals recorded at electrode F4. AMI calculation was as follows:


(3) $$\mathrm{AMI}=\frac{\alpha_{\mathrm{AR}}-\alpha_{\mathrm{AW}}}{\alpha_{\mathrm{AR}}+\alpha_{\mathrm{AW}}}$$

where $\alpha_{\mathrm{AR}}$ and $\alpha_{\mathrm{AW}}$ represent FFT-produced alpha frequency band powers of one of the subjects’ attention-related leads for the right and wrong selection trials, respectively. FFT uses the “fft()” function in the SciPy library, and the parameters are the default values. The number of Attention state trials exceeds that of Inattention state trials, to ensure the test validity; when the offline processing is performed, a number of Attention state trials equal to that of Inattention state trials will be randomly extracted from the data of one set.

TBR index is shown in the Equation 4. $E_{\text{Theta}}$ is the energy of theta rhythm and $E_{\text{Beta}}$ is the energy of beta rhythm. The rhythmic energy is calculated by wavelet transform. Wavelet transform uses the “Waveletpacket()” function in pywt library, the default value of parameter “wavelet” is “db4,” and the default value of parameter “maxlevel” is 8. A decrease in the ratio represents attention, and an increase in the ratio represents inattention.


(4) $$\mathrm{TBR}=\frac{E_{\text{Theta}}}{E_{\text{Beta}}}$$

To statistically validate differences between attention conditions in time–frequency spectra (ERSP), we employed cluster-based permutation testing, a non-parametric statistical approach robust to multiple comparisons (Maris and Oostenveld, 2007). Clusters were formed from adjacent time–frequency bins exceeding *p* < 0.05 in paired *t*-tests, with cluster masses defined as the sum of t-values. A null distribution of maximum cluster masses was obtained over 1,000 permutations of within-subject condition labels, and observed clusters exceeding the 95th percentile (corrected *p* < 0.05) were deemed significant.

Paired-samples *t*-tests were conducted to compare ERD, AMI, and TBR between Attention and Inattention conditions across subjects. Normality of data was verified using the Shapiro–Wilk test before performing parametric tests. Pearson’s correlation coefficients (r) were calculated to assess relationships between ERD and attentional measures (AMI and TBR). Statistical significance was set at a *p*-value of < 0.01 to correct for multiple comparisons and control the false discovery rate. All statistical computations were rigorously documented and implemented in Python (version 3.8.10), utilizing libraries including NumPy, SciPy, Matplotlib, and PyWavelets, ensuring full reproducibility of results.

## 4 Results

### 4.1 Time- and frequency-domain analysis and results

In the international 10–20 system, the Cz electrode is located at the vertex of the scalp—roughly halfway between the nasion and inion—and is widely recognized as being sensitive to neural activity originating in the sensorimotor cortex. However, it is important to note that the electrode measures only scalp-level electrical activity and does not directly localize specific cortical structures. Without applying source localization techniques, any inference regarding the precise underlying cortex should therefore be made with caution. In this study, we used ERSP values recorded at Cz to generate time–frequency maps (1–30 Hz) using Python.

**Figure 2:**
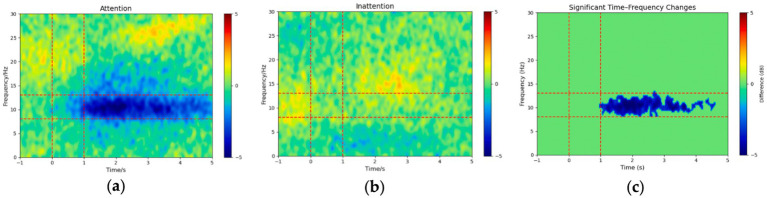
Grand-average ERSP (dB) from −1 to 5 s and 1–30 Hz under **(a)** Attention and **(b)** Inattention conditions, and **(c)** the cluster-based permutation-tested difference (Attention − Inattention; *p* < 0.05, corrected; non-significant bins zeroed out). Dashed red lines mark the *α* band (8–13 Hz) and key-press window (0–1 s).

To investigate the spectral dynamics associated with the “attention” and “inattention” conditions, we computed the grand-average ERSP across all 14 participants. This yielded two time–frequency representations: Figure 2a for the attention condition and Figure 2b for the inattention condition, each spanning the 1–30 Hz frequency range over a − 1 to 5 s time window. As shown in Figure 2a, the attention condition exhibits a pronounced negative power (blue region) in the alpha band during the motor imagery period, indicating a stronger ERD. In contrast, the inattention condition (Figure 2b) is more diffuse and does not display a comparably pronounced negative power shift in the alpha band.

To assess whether the observed differences between the Attention and Inattention conditions were statistically significant, we applied a cluster-based permutation test. Our analysis revealed a significant cluster in the alpha band, confirming that the difference in ERSP between the Attention and Inattention conditions is statistically robust (*p* < 0.05, corrected). In Figure 2c, only those time–frequency bins comprising that significant cluster are displayed (all other bins set to zero), highlighting a coherent *α*-band desynchronization emerging approximately 1 s and persisting throughout the motor imagery period. These results underscore the critical role of attentional modulation in facilitating cortical desynchronization and highlight the importance of incorporating attention-focused paradigms when investigating motor imagery. These findings suggest that under higher attentional demands, cortical activation in the alpha frequency range undergoes significant modulation. The group-level results align with our individual-level analyses, reinforcing the notion that attention can enhance ERD during lower-limb motor imagery tasks.

### 4.2 Frequency- and time-domain energy analysis and results

To further explore the ERD phenomenon under Attention and Inattention conditions in the alpha and beta bands, Python programming is used. After obtaining the ERSP value according to Equation 1, with the superposition of ERSP values in time domain, the brain electric energy change curves in frequency are obtained.

**Figure 3:**
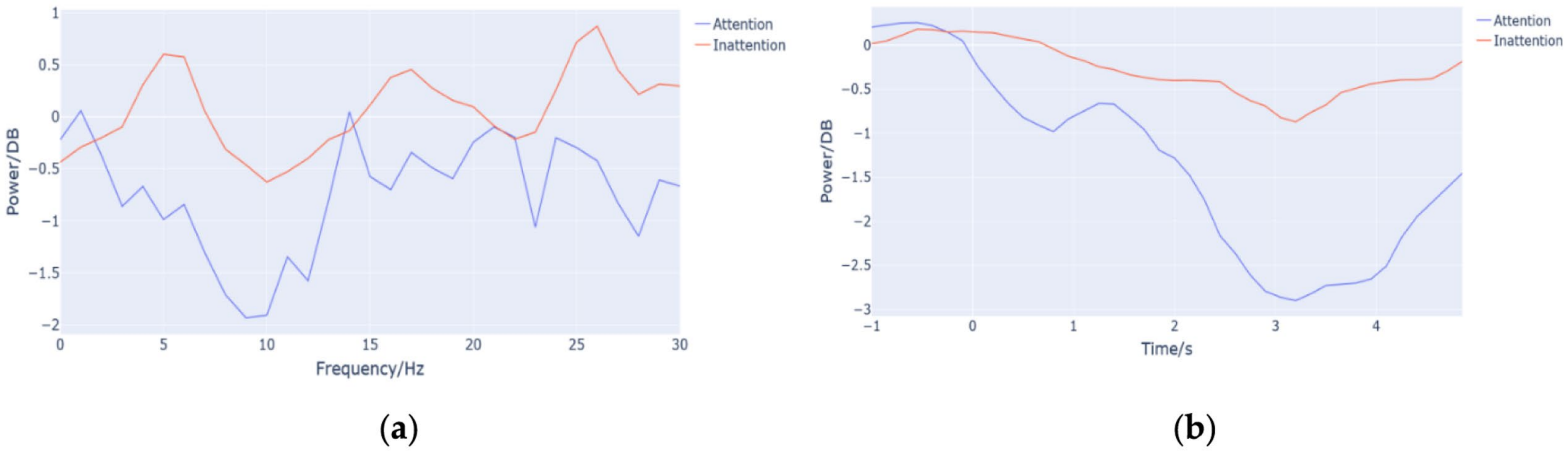
**(a)** Frequency-domain energy curve. The abscissa represents the frequency (Hz), and the ordinate represents the average value (DB) of brain energy in the corresponding frequency band of the 14 subjects in the time range of 1–5 s. **(b)** The time-domain energy curves. The abscissa represents the time (s), and the ordinate represents the average value (DB) of brain energy in the corresponding frequency band of the 14 subjects in the time range of 1–5 s.

Figure 3a shows average frequency-domain energy curves of the 14 subjects, and the energy value of high attention is lower than that of low attention in alpha frequency band. Figure 3b is the average time-domain energy curves of the 14 subjects, which was obtained by the superposition of ERSP values in the frequency domain, a method different from that used in Figure 3a. It can also be seen that the ERD phenomenon under Attention condition is more significant than that under Inattention condition.

### 4.3 Spatial domain analysis and results

The alert function is responsible for persistent attention. Sustained attention (or alertness) is necessary for long, tedious tasks. Although the attention neural network has not been fully understood, it seems that the scope mainly involves the right hemisphere of the brain (frontal and parietal lobe), including the right inferior parietal lobule, AG area, and thalamus area (Nimmy John et al., 2019).

**Figure 4:**
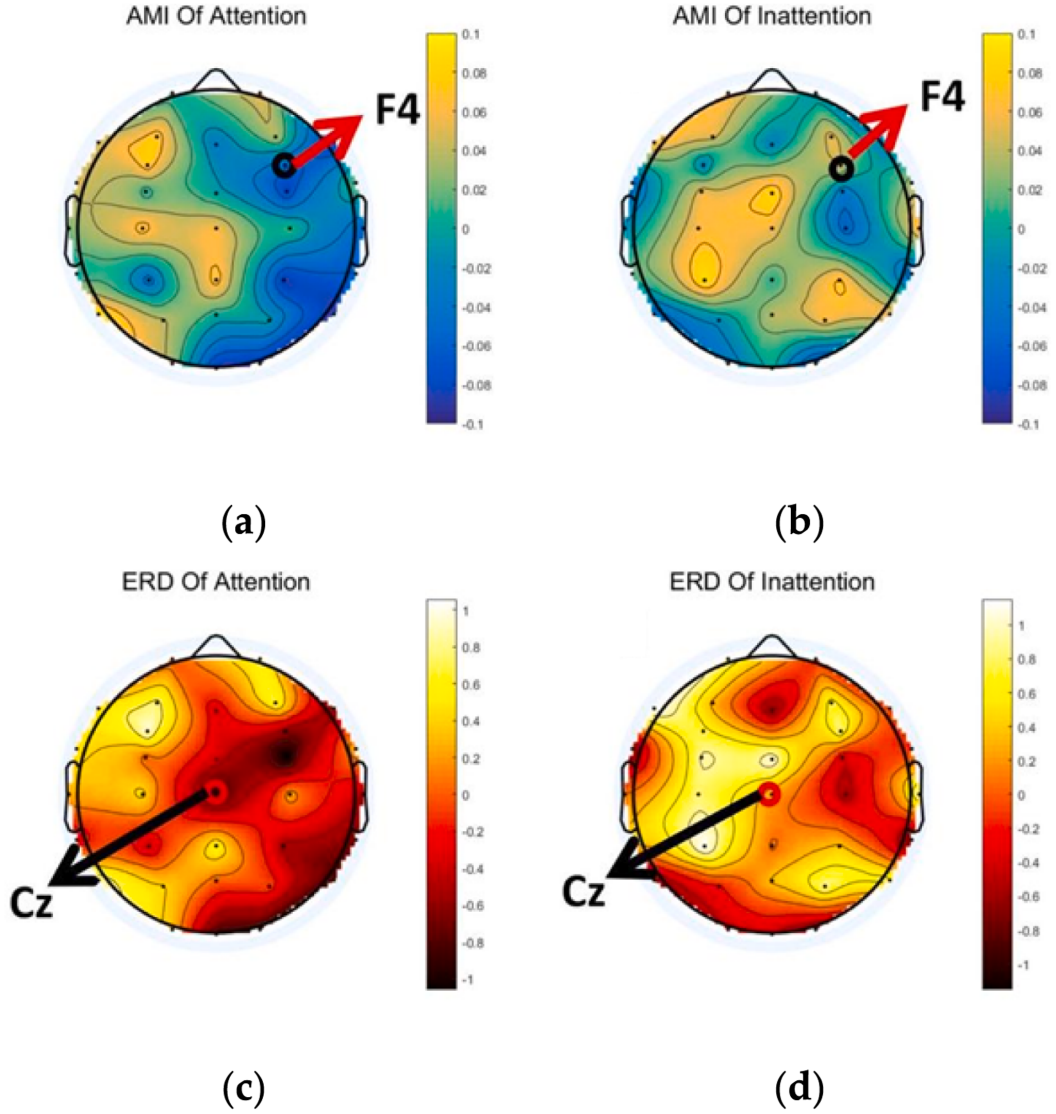
Averaged brain topographical map. **(a,b)** The AMI of Attention and Inattention and **(c,d)** the ERD of Attention and Inattention.

The AMI and ERD of 32 electrodes were computed and plotted via Python. Since F4 lies within the right-frontal “alert” network, AMI values at F4 are visibly lower under Attention than Inattention, indicating enhanced attentional engagement (Figures 4a,b). Similarly, ERD around Cz is markedly stronger under Attention, reflecting deeper *α*-band desynchronization during lower-limb motor imagery (Figures 4c,d).

To statistically confirm these observations, we extracted mean AMI and ERD values at each electrode and performed a cluster-based permutation test (1,000 permutations; initial threshold *p* < 0.05). A single significant cluster was identified over right-frontal sites (F4, FC4), with cluster mass = −12.8 (corrected *p* = 0.023), validating that α-band desynchronization under focused attention is reliably localized in this region.

### 4.4 ERD and AMI analysis and results

From the spatial domain analysis, this study mainly selects the mean AMI from EEG data of F4 to evaluate attention state according to Equation 3 and the ERD mean value of each subject’s Attention and Inattention on the alpha frequency bands was obtained from EEG data of Cz according to Equation 2.

The statistical analysis methods were used to obtain the average of the total data of the 14 subjects’ value (mean), *p*-value of paired *t*-test. It can be seen from Table 1 that the mean of the average ERD of all subjects’ Attention is lower than that of Inattention (−1.7700 < −0.0094), which shows that the ERD phenomenon of Attention is more significant than that of Inattention.

**Table 1:** ERD mean value and AMI.

Subjects	Attention		Inattention	
	ERD	AMI	ERD	AMI
S1	−2.0164	−0.0573	−1.5252	−0.0703
S2	−2.3600	−0.0688	−1.6173	−0.0759
S3	−3.4486	−0.0974	−1.1518	−0.0694
S4	−0.8000	−0.0205	0.1548	0.0021
S5	−2.4723	−0.0613	1.1942	0.0804
S6	−0.1843	0.0274	−0.8234	−0.0154
S7	−1.1349	−0.0385	0.6306	0.0469
S8	−0.8348	−0.0231	0.5188	0.0365
S9	−0.0784	0.0157	−0.3884	−0.0046
S10	0.7046	0.0486	0.5164	0.0344
S11	−4.3186	−0.0986	0.6518	0.0476
S12	−3.1437	−0.0894	0.5769	0.0371
S13	−2.7763	−0.0653	0.5098	0.0342
S14	−1.9164	−0.0591	0.6209	0.0466
Mean	−1.7700	−0.0419	−0.0094	0.0093
SDT	1.3849	0.0446	0.8816	0.0483
*r*	0.9641		0.9495	
*p*	2.8310*10^−8^		2.1229*10^−7^	

Mean stands for the average of 14 subjects in each column, and the *p*-value corresponds to the correlation coefficient between ERD and AMI in the state of Attention and Inattention, respectively.

As shown in Table 1, on the premise of Attention (*p* = 2.8310*10^−8^ < 0.01), the mean of ERD is significantly positively correlated with AMI, and AMI can be used as a quantitative index to measure the degree of attention.

### 4.5 TBR offline analysis and results

**Figure 5:**
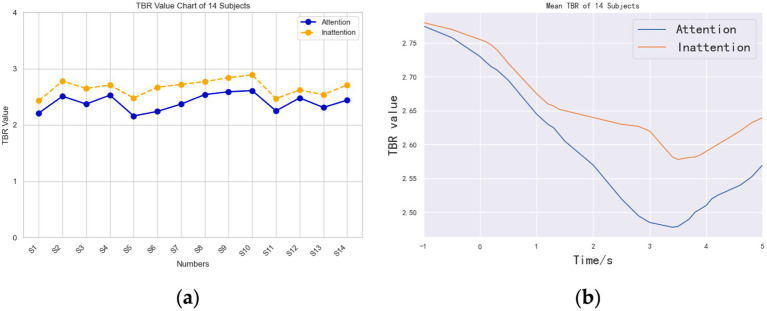
TBR index curves of Attention and Inattention. **(a)** TBR value chart of 14 subjects. **(b)** Average time-domain TBR curves of the 14 subjects.

Figure 5a shows the 1- to 5-s TBR values in the two different states of “Attention” and “Inattention.” In Figure 5a, “Attention” and “Inattention” curves represent the mean values of the experimental results of each subject in a high or low attention state.

The value of TBR of all subjects under the condition of Attention is less than that under the condition of Inattention. Since the value of TBR is negatively correlated with the degree of attention, that is, the lower the value, the higher the degree of attention, so the attention state under Attention condition is better than that under Inattention condition. In terms of correlation, it shows that this paradigm is helpful to improve the attention state of motor imagery to a certain extent.

Figure 5b is obtained by offline data processing, and it is the average time-domain TBR curves of the 14 subjects. Unlike the real-time coverage of online data, offline data can produce continuous TBR indicators every second, which can further refine the changes of TBR. As can be seen from Figure 5b, the TBR index in the Attention state is less than that in the Inattention state within the −1–5 s time range, which shows that attention is more focused on the former state than in the latter state.

A paired-samples *t*-test on mean 1–5 s TBR confirmed a significant reduction under Attention (2.55 ± 0.15) versus Inattention [2.58 ± 0.17; *t*_(13)_ = 5.12, *p* = 2.4 × 10^−5^, Cohen’s d = 1.37], demonstrating TBR’s validity as an offline index of attentional state.

The statistical analysis methods were used to obtain the average of the total data of 14 subjects (mean) and the *p*-value of paired *t*-tests. As shown in Table 2, a *p*-value of < 0.01 in both attention states indicates that the mean ERD is significantly positively correlated with mean TBR, supporting TBR as a quantitative ERD index.

**Table 2:** ERD and TBR for the subjects performing motor imagination.

Subjects	Attention		Inattention	
	ERD	TBR	ERD	TBR
S1	−2.0164	2.3812	−1.5252	2.3041
S2	−2.3600	2.3354	−1.6173	2.3956
S3	−3.4486	2.3867	−1.1518	2.3754
S4	−0.8000	2.7402	0.1548	2.7892
S5	−2.4723	2.4687	1.1942	2.5904
S6	−0.1843	2.4665	−0.8234	2.5572
S7	−1.1349	2.6942	0.6306	2.7405
S8	−0.8348	2.5445	0.5188	2.4922
S9	−0.0784	2.6741	−0.3884	2.7631
S10	0.7046	2.8144	0.5164	2.8642
S11	−2.1853	2.3856	−0.7518	2.4522
S12	−1.2965	2.5771	0.4769	2.6837
S13	−0.5537	2.6593	−0.2098	2.6508
S14	−1.9164	2.5884	−1.4405	2.4663
Mean	−1.7700	2.5511	−0.3154	2.5803
SDT	1.3849	0.1459	0.8863	0.1667
*r*	0.7658		0.6987	
*p*	0.0014		0.0054	

ERD represents the mean value of 1–5 s ERD energy for the Subject. TBR represents the mean value of 1–5 s TBR. Mean stands for the average of 14 subjects in each column, and the *p*-value corresponds to the correlation coefficient between ERD and TBR in the state of Attention and Inattention, respectively.

### 4.6 Online time-domain analysis and results

Due to the poor real-time performance of AMI indicators, AMI is not suitable to reflect the attention state during the implementation of motor imagery in real time, while TBR can be used as the index of real-time evaluation according to Equation (4). The program is set to read and process 1-s data in the buffer each second. At the same time, because the data are discontinuous, the TBR index of online data can only be calculated once a second.

**Figure 6:**
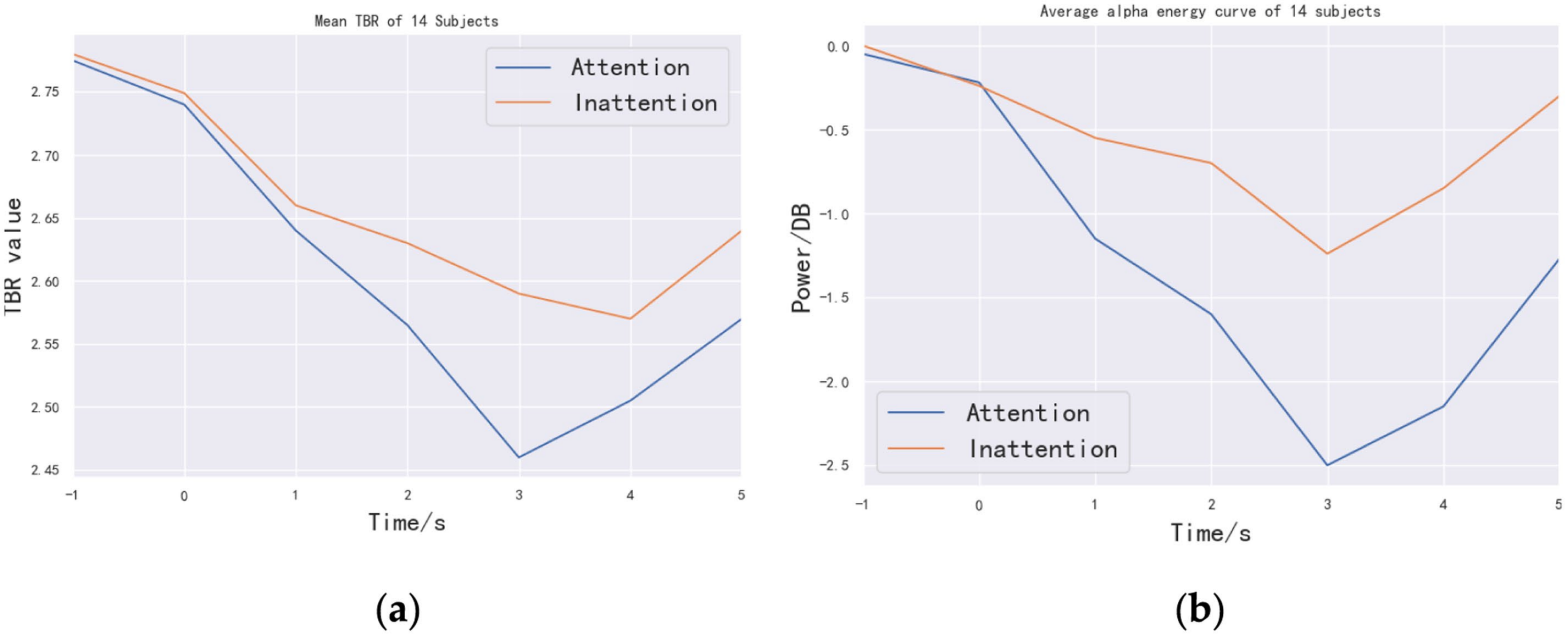
Time-domain curves of mean diagram of TBR and energy: **(a)** line chart of TBR mean of the 14 subjects and **(b)** average time-domain alpha energy curve of the 14 subjects. The abscissa of the two graphs is time. The purpose is to observe the trend of TBR value and power over time.

Figure 6a shows the mean value of TBR of the 10 subjects over time. In the range of 1–3 s, TBR index decreases in both Attention and Inattention states, indicating that attention is focused. Within the time range of performing motor imagery (1–5 s), the TBR value of Attention is less than that of Inattention, which shows that the TBR index can well distinguish the two attentional states. Figure 6b is a graph of the average alpha rhythm energy of the 14 people over time. Consistent with Figure 6a, the energy of alpha rhythm shown in Figure 6b decreases in the range of 1–3 s, indicating that there is an obvious ERD phenomenon. The TBR index and ERD phenomenon have some consistency in time domain, which indicates that the effect of motor imagery and attention degree (TBR) has certain correlation. At the same time, in the range of 1–5 s, the changing trend of TBR in Attention state is consistent with that of alpha rhythm energy, indicating that TBR index can be used as a real-time judging criterion.

To statistically validate online discrimination, paired-samples *t*-tests were performed at each second from 1 s to 5 s with Bonferroni correction (*α* = 0.01/5). Significant TBR reductions under Attention were observed at 2 s [*t*_(13)_ = 5.28, corrected *p* = 4.3 × 10^−5^], 3 s [*t*_(13)_ = 5.45, corrected *p* = 2.8 × 10^−5^], and 4 s [*t*_(13)_ = 4.92, corrected *p* = 6.1 × 10^−5^], closely matching concurrent α-energy decreases and confirming TBR’s real-time sensitivity.

## 5 Discussion

MI as a key technique for enhancing motor skill acquisition and neural adaptation training, via the mental simulation of actions, has attracted considerable attention in the fields of exercise science and brain–computer interfaces (BCI) (Burianová et al., 2013). EEG-based MI BCI systems offer novel approaches to optimizing motor performance by decoding neurophysiological features such as ERD and the AMI (Agosti and Sirico, 2020). In this study, we propose a novel experimental paradigm that combines a key-press task to modulate attentional states with the real-time advantages of the TBR to systematically investigate the relationship between attention levels and neural activity (ERD/AMI) during lower-limb MI in healthy adults. Our findings confirm the practical utility of TBR in lower-limb MI, complementing the results of Gu et al. (2020) on the lateralization of beta rhythm networks. However, compared with upper limb MI studies (Zhang et al., 2016), the ERD intensity during lower-limb tasks is generally lower, which may be related to differences in motor cortex activation patterns (Grilc et al., 2024). Moreover, the real-time performance of TBR surpasses that of traditional neurofeedback indicators, supporting its potential application in motor training (Cheng et al., 2024).

In comparison with previous research, our results align with the conclusions of Gabbard and Fox (2013) regarding the impact of attentional states on MI quality and further support (Jeunet et al., 2020) view on the limitations of traditional offline indicators in real-time monitoring. Unlike most studies that focus solely on offline data analysis, this research implements real-time monitoring of attentional states (Souza and Naves, 2021) through the introduction of the TBR index. We acknowledge that although online systems offer significant advantages in terms of immediate feedback, there remains room for improvement in signal precision. The discrepancies between online and offline experimental results mainly stem from differences in the real-time processing and accuracy of data. Offline experiments allow for the use of larger datasets for filtering and post-processing, thereby yielding more stable ERD and AMI indices; in contrast, online experiments are constrained by the speed of real-time data transmission and processing, which may result in slight fluctuations in these indices. Nevertheless, we contend that despite the presence of some noise, the real-time TBR index can provide immediate feedback that is of practical value for rapidly adjusting attentional states during motor training (Tan et al., 2024).

Furthermore, some studies have suggested that low gamma waves may carry information regarding higher-order cognitive states (Grassini et al., 2019) and emotional changes—a topic that we believe warrants further investigation in future research (Saibene et al., 2023). Importantly, our spatial analysis confirmed a right-frontal *α*-band cluster (F4, FC4; *p* = 0.023) via cluster-based permutation testing, validating the topographic specificity of attentional ERD. In addition, we have also reached cooperation with Tianjin Union Medical Center. The ethics agreement document number is TJUMC-2022-C05. In the future, if conditions permit, data will be collected for experimental research with the consent of patients. Other EEG-based indicators have also shown potential in monitoring cognitive states and attention levels, such as the Engagement Index (EI) and the Mental Workload Index (MWI). These indices may complement each other in capturing different aspects of attention and cognitive load and thus warrant further exploration in future research.

## 6 Conclusion

This study revealed that attention state significantly affects the neural efficacy of lower-limb motor imagery by regulating ERD/AMI and confirmed that TBR can be used as a real-time attention monitoring indicator. The results demonstrated that subjects exhibited more pronounced ERD under high attentional states, and a significant correlation was observed between the TBR index and the AMI, thereby confirming the feasibility and efficacy of TBR as a real-time attention metric. Cluster-based permutation tests further established that *α*-band ERD differences are statistically robust (corrected *p* < 0.05), and paired *t*-tests demonstrated significant offline TBR reductions [*t*_(13)_ = 5.12, *p* = 2.4 × 10^−5^] and online discrimination (Bonferroni-corrected *p* < 0.01). Our findings suggest that lower-limb motor imagery conducted under high attentional states can improve signal quality and enhance the overall effectiveness of motor imagery. In summary, this study provides a novel real-time attention tracking method for motor skill training and neural adaptation training, thereby extending the neuroscientific foundation for optimizing motor performance.

## Data Availability

The datasets presented in this article are not readily available because the data that support the findings of this study are not openly available due to reasons of sensitivity and are available from the corresponding author upon reasonable request. Requests to access the datasets should be directed to PL, lph1973@tju.edu.cn.
