# Supplementary material for: Influence of attentional state on EEG-based motor imagery of lower limb
Source: Front Hum Neurosci. 2025 May 21;19:1545492. doi: 10.3389/fnhum.2025.1545492 (PMC12133726; doi:10.3389/fnhum.2025.1545492)
Supplement: Supplementary file 1 [file Supplementary_file_1.docx]

Supplementary Material

# Supplementary Figures

**
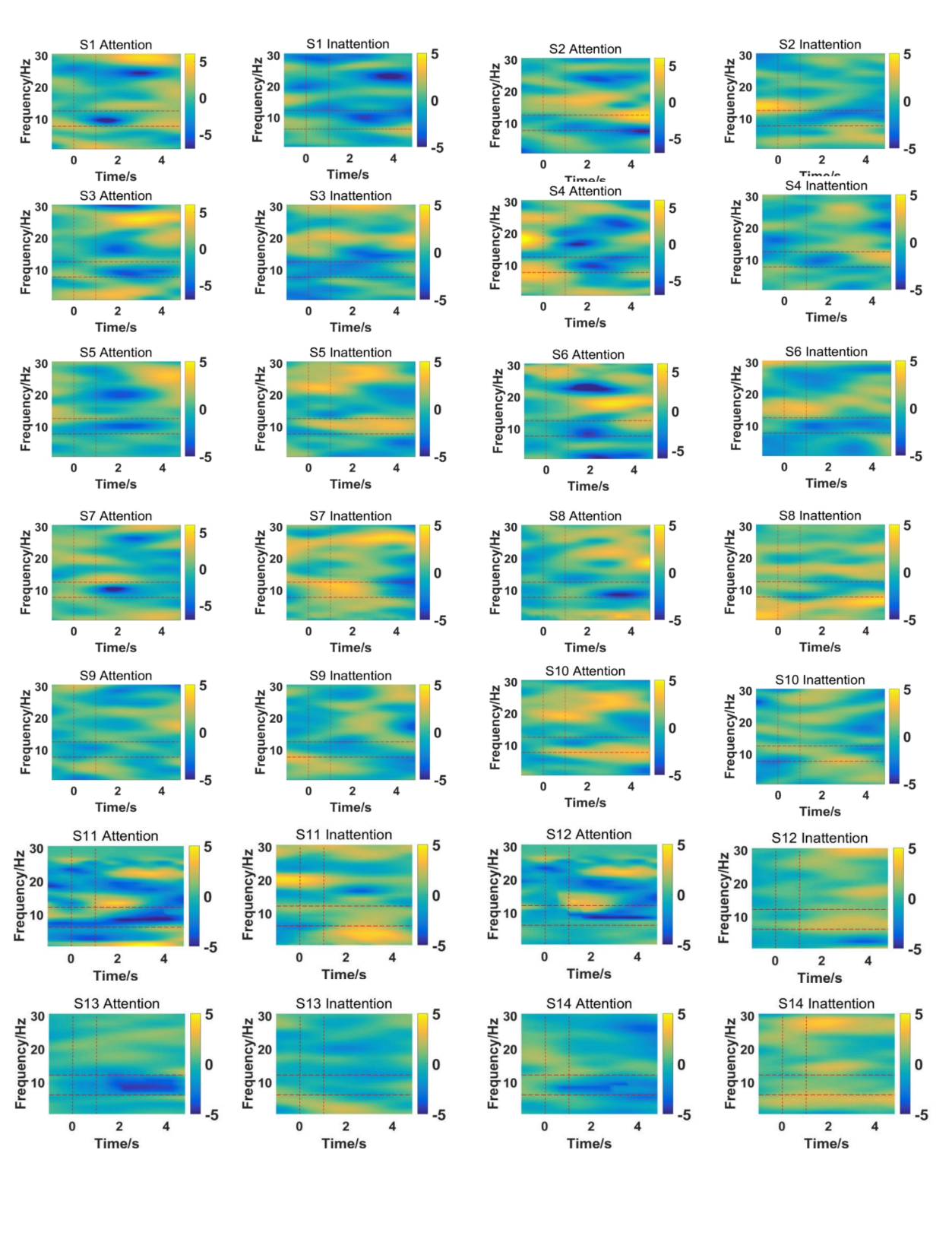
**

**Supplementary Figure 1.** The time-frequency diagrams of all the subjects during Attention and Inattention at the electrode position Cz.
